# Plant-Derived (Poly)phenols and Their Metabolic Outcomes: The Pursuit of a Role for the Gut Microbiota

**DOI:** 10.3390/nu14173510

**Published:** 2022-08-26

**Authors:** Perla Lopes de Freitas, João Paulo Nascimento Miranda, Lucas Martins França, Antonio Marcus de Andrade Paes

**Affiliations:** 1 Laboratory of Experimental Physiology, Department of Physiological Sciences, Biological and Health Sciences Center, Federal University of Maranhão, São Luís 65080-805, MA, Brazil; 2 Health Sciences Graduate Program, Biological and Health Sciences Center, Federal University of Maranhão, São Luís 65080-805, MA, Brazil

**Keywords:** prebiotics, (poly)phenols, gut microbiota, host metabolism, metabolic disorders

## Abstract

Plant-derived (poly)phenolic compounds have been undoubtedly shown to promote endocrine homeostasis through the improvement of diverse metabolic outcomes. Amongst diverse potential mechanisms, the prebiotic modulatory effects exerted by these compounds on the gut microbiota have supported their nutraceutical application in both experimental and clinical approaches. However, the comprehension of the microbiota modulatory patterns observed upon (poly)phenol-based dietary interventions is still in its infancy, which makes the standardization of the metabolic outcomes in response to a given (poly)phenol a herculean task. Thus, this narrative review sought to gather up-to-date information on the relationship among (poly)phenols intake, their modulatory effect on the gut microbiota diversity, and consequent metabolic outcomes as a supportive tool for the future design of experimental approaches and even clinical trials.

## 1. Introduction

In 1991, when Lynn Margulis first introduced the term “holobiont”, he meant to comprise the elementary biological entity formed by a sole host and a single inhabiting symbiont [1]. Thenceforth, it has become undisputedly clear that the human body and all the microbial communities that inhabit its surface and cavities constitute a genuine holobiont harboring a 33-million-gene hologenome whose expression results in complex interactions that shape all aspects of human biology [2,3,4]. Among the various microbial niches in the human body, the broader amount and diversity of symbiotic microorganisms is found in the intestinal colon with bacterial density ranging from 10^8^ to 10^11^ bacteria per gram of wet stool [5,6]. Nevertheless, the first body of evidence suggesting a modulatory role for gut microbiota on host energy balance and metabolism arose only 15 years ago [7,8,9].

The gut microbiota comprises a range of micro-organisms, such as bacteria, fungi, archaea, and viruses, with prevalence of bacteria from four main phyla: Firmicutes, Bacteroidetes, Actinobacteria, and Proteobacteria [10]. A fetal gastrointestinal tract contains no detectable microbiota [11], supporting the assumption that gut colonization begins only upon birth. However, it promptly undergoes an exponential expansion during the first 3 years of life, when it reaches levels and diversity comparable to adults [12]. Most of the time, these micro-organisms live in symbiosis with their host. However, diverse factors such as breastfeeding length, consumption of unhealthy diets, and self-guided use of antibiotics may alter the gut microbiota composition and favor the rise of pathogenic microbial species responsible for triggering dysbiosis [13,14]. The current evidence strongly supports a close relationship between gut dysbiosis and the pathogenesis of obesity, type 2 diabetes mellitus (T2DM), and metabolic syndrome (MetS) as well as other metabolic disorders [15,16].

In the meantime, huge evidence has also supported probiotic and prebiotic-based dietary interventions as complementary ways to modulate the gut microbiota in order to prevent or treat the aforementioned metabolic disorders [17,18,19]. By definition, probiotics are live micro-organisms that, when administered in adequate amounts, confer a health benefit on the host [20]. Meanwhile, prebiotics are substrates selectively utilized by host micro-organisms to confer a health benefit as well [21]. Among the latter, the nondigestible polysaccharides fructans and galactans, polyunsaturated fatty acids, and (poly)phenols have attracted much interest because of their capacity to drive gut bacteria metabolism toward the production of metabolites eventually responsible for health outcomes on the physiology of the host [22,23,24].

Plant-derived (poly)phenols are prebiotics of particular importance because of their presence in most dietary patterns—being found in vegetables, fruits, as well as their byproducts, such as chocolate, coffee, wine, and tea [25]—which account for a daily consumption estimated at, at least, 1 g per day [26]. However, roughly 95% of the ingested (poly)phenols are not absorbed and reach the distal colon [27], where they play an important role as substrate for colonic microbiota metabolism [28]. As a result, increased concentrations of (poly)phenols in the colonic lumen are thought to drive the growth of bacteria pertaining to the Bacteroidetes phylum, which modulate the gut microbiota composition toward a healthier mix of microbes [17,18,28].

Currently, it is consensually accepted that most metabolic disorders are associated with changes in the microbiota at the phylum level, resulting in an increase in the Firmicutes/Bacteroidetes ratio [29]. However, microbiota modulatory patterns observed upon (poly)phenol-based dietary interventions are far from consensual, either in rodents or human, which make the standardization of the metabolic outcomes in response to a given (poly)phenol a herculean task, particularly under a translational perspective. Thus, this narrative review sought to scrutinize the currently available information to settle the debate concerning the possible intermediating role that the gut microbiota plays in the metabolic outcomes promoted by (poly)phenols intake as a necessary instrument for the future design of experimental approaches and clinical trials.

## 2. Gut Microbiota Development from Early-Life Colonization to Dysbiosis-Related Metabolic Disorders

Since the first postulations made by the French pediatrician Henry Tissier over one century ago, it has been assumed that initial gut colonization only occurs upon birth because of the sterile environment provided by the placental barrier [30]. This assumption was recently corroborated by the demonstration that no microbial signal was detected in fetal meconium by 16S ribosomal RNA gene sequencing [11], contradicting previous reports of maternal microbial species detection in the meconium [31] and amniotic fluid [32]. Therefore, labor represents the foremost way of vertical microbiota transmission, while the type of birth strongly impacts the microbial diversity of the emerging microbiota of the offspring. Newborns exposed to vaginal microbes during delivery present microbiome rich in *Lactobacillus* and *Prevotella* spp. [33]. On the other hand, the microbiome of those born via C-section is more prevalent in *Staphylococcus*, *Corynebacterium*, and *Propionibacterium* spp., which resemble microbial species commonly found in the skin microbiota [34,35].

The gut microbiota expands exponentially during the first 3 years of life to reach a microbial diversity comparable to adults [12]. In adults, the gut microbiota contains around 160 out of 1000–1150 distinct symbiont species, as demonstrated by the European Metagenomics of the Human Intestinal Tract Study (European MetaHIT, accessible at http://metahit.eu, accessed on 30 July 2022), which supports a wide interindividual variability of microbiota composition [36]. In terms of abundance, most species pertain to the phyla Firmicutes and Bacteroidetes, followed by Actinobacteria, Proteobacteria, and Verrucomicrobia [37,38]. However, the proportional ratio among these phyla widely varies within the lifetime of an individual [39] in response to diverse factors, such as nutritional and dietary patterns, breastfeeding length, and exposure to xenobiotics and environmental toxicants [13]. Interestingly, microbes from Firmicutes and Proteobacteria phyla have been shown to prevail in the gut of European children exposed to a Western diet, while African children fed a rural diet had higher prevalence of Bacteroidetes and Actinobacteria [40].

As a result, these factors may eventually disrupt the symbiotic host–microbiota relationship, leading to the triggering of gut dysbiosis [14]. Dysbiosis can be defined from different perspectives, such as the loss of commensals, loss of diversity, or surge of pathobionts, which have recently been subject of an interesting review [41]. Even though, since a metabolic perspective, gut dysbiosis might be defined as an imbalance between healthy and pathogenic microbial species that favors the release of the gram-negative membrane protein lipopolysaccharide (LPS), a key mediator of low-grade inflammation [14,42]. In turn, low-grade inflammation is well-characterized as a common risk factor for the onset of most metabolic disorders, particularly those affecting the metabolic quartet: liver, pancreas, white adipose tissue, and skeletal muscle [43,44,45].

The mechanisms interconnecting these apparently remote tissues started to be elucidated in a seminal study by Cani et al. [42] showing that mice fed a high-fat diet for 4 weeks presented with LPS plasma levels that increased by 2–3 times, which was associated to body weight gain, insulin resistance onset, and white adipose tissue dysfunction. Such findings led the authors to characterize LPS rising as the trigger factor of so-called metabolic endotoxemia [42]. Metabolic endotoxemia has also been demonstrated in genetically obese mice [46,47] and humans [48,49]. On the other hand, it has been recently demonstrated that LPS from certain bacteria, such as *Rhodobacter sphaeroides*, indeed, led to a metabolically beneficial endotoxemia characterized by improved adipose tissue insulin signaling and restored dysglycemia in obese mice [50].

Concerning microbiota patterns associated to metabolic disorders, early studies showed that obese rodents present with an increased abundance of Firmicutes phylum in parallel to a decreased abundance of Bacteroidetes [5,51], as well as *Akkermansia* species [52]. In humans, data have been controversial, with some reports finding the same pattern in rodents [53,54] or the very opposite [55,56]. Nevertheless, it has been accepted that metabolic disorders are associated with changes in the microbiota at the phylum level, which promote the increase in the Firmicutes/Bacteroidetes ratio [29] and alter the release of microbiota-derived metabolites, such as the short-chain fatty acids (SCFA) [22,23].

Once produced in the colonic lumen, the main SCFA—i.e., acetate, propionate, and butyrate—reach the bloodstream at different ranges [57]. At a cellular level, the SCFA bind to a set of G-protein-coupled receptors, namely, GPR41, GPR43, and GPR109a, which are ascribed as mediators of SCFA effects on host metabolism [58]. SCFA have also been shown to modulate the host metabolism through GPR-independent pathways, such as the inhibition of histone deacetylases [59]. However, a recent study demonstrated that the metabolic benefit promoted by SCFA derived from dietary fibers intake is larger than that promoted by dietary supplementation with pure SCFA [60], supporting the importance of prebiotics, such as nondigestible polysaccharides and plant-derived (poly)phenols, as sources of microbial metabolites able to modulate the host metabolism.

## 3. Modulatory Effects of Plant-Derived (Poly)phenols on the Gut Microbiota and Their Metabolic Outcomes

(Poly)phenols constitute a large group of over 8000 distinct phenolic compounds identified so far [61]. Given their wide occurrence in vegetables, fruits, and their byproducts, which are included in eventually all dietary patterns, (poly)phenols dietary intake is estimated at, at least, 1 g per day [26]. However, as their absorption is very limited, roughly 90–95% of the ingested (poly)phenols reach the colon unscathed [27]. At the colon lumen, (poly)phenols are deglycosylated, i.e., their glycoside bonds and heterocyclic backbone are broken down to generate absorbable bioactive metabolites [28], particularly short-chain fatty acids (SCFA) [62]. Comparing the two main phyla, bacteria from Bacteroidetes have been shown to express higher levels of glycan-degrading enzymes than Firmicutes [63], making it reasonable to suggest that higher colonic concentrations of (poly)phenols might benefit the metabolism and growth of Bacteroidetes more than Firmicute. As a result, the Bacteroidetes/Firmicutes ratio would be increased, leading to wider beneficial metabolic outcomes.

Thus, to verify the reasonability of this rationale, the following descriptors combination was used to search Pubmed and retrieve over 500 studies published between 2008 and 2022: “gut microbiota and (poly)phenols”. Three criteria for inclusion were applied: (a) the use of pure, isolated (poly)phenols; (b) the characterization of the gut microbiota modulation upon (poly)phenol treatment; and (c) the assessment of in vivo metabolism-related outcomes. Most of the studies retrieved were in vitro or based on the assessment of plant extracts, which did not make clear the compound(s) responsible for the described effects. As shown in Table 1, only 32 studies using purified compounds, which pertained to stilbenoids, flavonoids, and capsaicinoids classes of (poly)phenols, were considered appropriate.

Resveratrol is the leading (poly)phenol in the microbiota-based studies herein described (Table 1). Chemically, resveratrol is a phenylalanine-derived (poly)phenol pertaining to a large, structurally diverse class of oligomeric stilbenoids. [96,97]. An inaugural study by Larrosa et al. (2009) demonstrated that a 25-day treatment with a low resveratrol dose (1 mg/kg/day) increased *Lactobacilli* and *Bifidobacterium*, as well as avoided the increase in *Enterobacteria* upon colitis induction [64]. Dietary supplementation with resveratrol at a much higher dose (400 mg/kg/day) for high-fat high-sugar (HFHS)-fed mice for 8 weeks increased the Bacteroidetes/Firmicutes ratio, particularly by increasing the growth of *Bacteroides* and *Parabacteroides* genera [68]. However, administration of resveratrol at the same dose (400 mg/kg/day) for 16 weeks to high-fat diet (HFD)-fed mice contrarily increased the relative abundances of bacteria pertaining to Firmicutes, Proteobacteria, and Verrucomicrobia phyla [70].

More recently, it has been shown that an 8-week supplementation with resveratrol (30 mg/kg/day) or its 2-methoxy derivative pterostilbene (15 or 30 mg/kg/day) barely changed gut dysbiosis in high-fat high-fructose (HFHF)-fed mice, despite preventing steatohepatitis development [75]. Of note, pterostilbene (15 mg/kg/day for 6 weeks) had been previously reported to reduce the abundance of Firmicutes phylum on the gut of obese Zucker (*fa/fa*) rats [76]. Still, resveratrol supplementation to HFD-fed rats at 10 mg/kg/day [74] or to HFHS-fed mice at 400 mg/kg/day [68] improved glucose metabolism and insulin sensitivity in a very similar manner, despite exerting opposite effects on the Bacteroidetes/Firmicutes ratio, which supports that resveratrol is capable of improving the metabolic profile despite the lack of a demonstrable microbiota modulation pattern.

Few studies have assessed the effects of resveratrol on human microbiota (Table 1). A recent pilot, randomized, placebo-controlled clinical trial with 28 obese men receiving 2 g/day trans-resveratrol for 30 days found an substantial modulation in the abundance of several taxa but with metabolic outcomes limited to a slight improvement of glucose tolerance, an effect restricted to Caucasian subjects [93]. On the other hand, a randomized, double-blind, placebo-controlled trial including 37 overweight and obese men and women receiving a combination of epigallocatechin-3-gallate (EGCG) and resveratrol (282 and 80 mg/day, respectively) for 12 weeks showed that (poly)phenols supplementation significantly decreased Bacteroidetes and tended to reduce *Faecalibacteriuim* *prausnitzii* in men but not in women [94]. These studies importantly unveil the matter of sexual and ethnical influences on the individual response to (poly)phenols intake, as well as the possible mutual antagonism among distinct phenols, which might be responsible for the limited metabolic benefits observed in the latter. Regardless of flavonoids being one of the most studied groups of (poly)phenols, we found scarce in vivo studies devoted to this phytochemical class (Table 1). EGCG, a flavan-3-ol compound, seems to be particularly active against the Firmicutes phylum. EGCG administration at 200 or 400 mg/kg/day for 4 weeks to chow-fed rats strongly decreased the relative abundances of distinct *Clostridium* clusters [77]. Similarly, its administration at a very high dose of 3000 mg/kg/day for 2 or 10 days almost abolished the colonies of the *Clostridium* cluster IV and *Clostridium* cluster XIVa in stools from lean mice at both times [78]. In both studies, EGCG had no impact on the metabolic profile of the lean chow-fed rodents. On the other hand, in HFD-fed mice, EGCG (33 mg/kg/day for 8 weeks) increased relative abundances of *Clostridium* genus but decreased other Firmicutes families, such as *Lachnospiraceae* and *Ruminococcaceae,* resulting in a reduced Firmicutes/Bacteroidetes ratio which was associated with the reversal of body weight gain and liver steatosis [79].

In an elegant study by Porras et al. [80], 16-week supplementation of a high-fat diet with 0.05% quercetin prevented the development of HFD-induced dysbiosis in mice. Of note, quercetin administration did not change the relative percentage of Firmicutes phylum, although it decreased Proteobacteria and increased Bacteroidetes, promoting a significant increment of the Bacteroidetes/Firmicutes ratio. This fact was allegedly associated with the profound metabolic improvement observed in the quercetin-treated mice, as shown in Table 1, via the recovery of intestinal barrier integrity and reversal of LPS-induced metabolic endotoxemia [80]. Other studies using hesperetin in chow-fed rats and theaflavins in diabetic *db*/*db* mice found rather conflicting data. Hesperetin decreased the relative abundance of the *Clostridium* subcluster XIVa but increased those of clusters IV and XVIII [81], whereas theaflavins decreased bacteria abundances in spite of the phyla they belong to [82].

Phenolic compounds harboring a sole aromatic ring are also capable of modulating host microbiota. Dietary supplementation with 6 mg/kg/day sinapine, an anticholinesterase phenolic acid, for 12 weeks decreased the Firmicutes/Bacteriodetes ratio, particularly by reducing the relative abundances of *Lachnospiraceae*, *Erysipelotrichaceae*, *Peptostreptococcaceae* families, meanwhile promoting a whole metabolic improvement [83]. In turn, Wang et al. [84] used an animal model of metabolic disruption caused by exposure to fine particulate matter to demonstrate that oral administration of hydroxytyrosol (50 mg/kg/day) for 4 weeks only slightly improved the dysbiosis by enhancing Bacteroidetes and reducing Firmicutes and Actinobacteria abundances. In the meantime, the same compound at the same dose administered to HFD-fed mice for 8 weeks did not alter the Firmicutes/Bacteroidetes ratio [85] (Table 1). These studies importantly suggest that distinct environmental factors disturb the gut microbiota homeostasis in different ways, making a particular (poly)phenol more or less effective in accordance to the dysbiosis inducer.

Capsaicinoids constitute a particular class of (poly)phenols containing a sole aromatic ring that, despite conflicting reports on their classification as alkaloids or phenolic compounds, have been assessed for different metabolic purposes [98], including modulation of the gut microbiota [99]. Capsaicin, the prototypic member of this class, has also shown conflicting results depending on the animal model applied and has been shown to modulate gut microbiota in both rodents [86,87,90] and humans [95]. Administration of capsaicin (2 mg/kg/day) for 12 weeks to HFD-fed mice increased the relative abundances of *Ruminococcaceae* and *Lachnospiraceae* families, both pertaining to the Firmicutes phylum, with an important decrease in LPS-producing S24-7 family, which is clustered in the Bacteroidetes phylum [86]. Using a five-fold higher dose of capsaicin (10 mg/kg/day, 9 weeks), Shen et al. [87] found a general increase in the relative abundances of different phyla in the feces of HFD-fed mice.

On the other hand, administration of capsaicin to genetically obese mice showed a quite opposite modulatory response (Table 1). Dietary supplementation of *ob/ob* mice with 0.01% or 0.02% capsaicin for 6 weeks consistently increased the Firmicutes/Bacteroidetes ratio in both doses [88]. Moreover, 0.01% capsaicin supplementation in *db/db* mice for 8 weeks prevented the increase in Firmicutes-pertaining bacterial genera with no change in those from the Bacteroidetes phylum [89]. The *ob/ob* mice have been shown to present a relative abundance of Firmicutes, 35% higher than Bacteroidetes, while *db/db* mice presented proportional abundances between these two phyla [100]. Such differences were attributed to their different genetic backgrounds [100], which might interfere with the response of microbiota to capsaicin.

Of note, capsaicin is a naturally occurring agonist of transient receptor potential vanilloid-1 (TRPV1) receptor [101], whose deletion has been associated to the onset of local inflammation and systemic progression toward sepsis [102]. On this account, oral administration of capsaicin (2 mg/kg/day, 12 weeks) to female TRPV1^−/−^ mice strongly decreased the relative abundances of diverse genera and families pertaining to the endotoxemia-associated Proteobacteria phylum, while also reducing body weight, food intake, as well as improved diverse markers of the glucose-insulin axis function (Table 1) [90]. Thus, modulatory effects of capsaicin on gut microbiota of TRPV1^−/−^ mice consistently support a TRPV1-independent mechanism of action for this compound.

**Figure 1:**
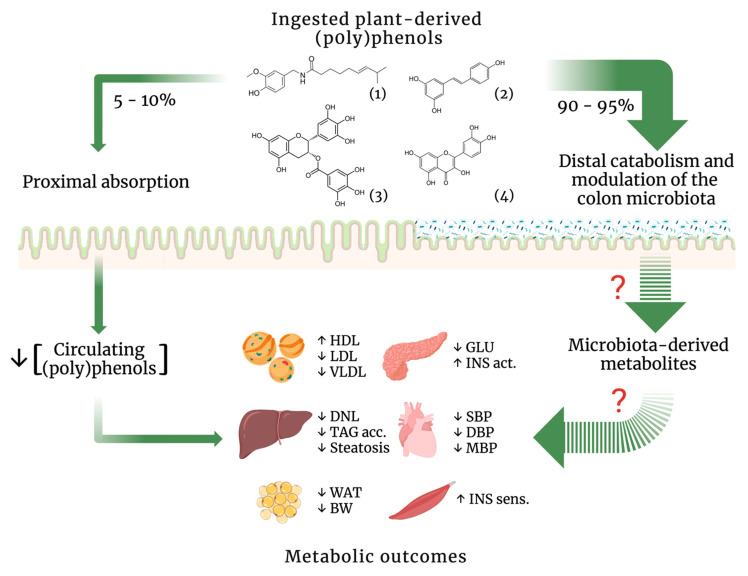
Biological fate of ingested plant-derived (poly)phenols and their metabolic outcomes. Schematic diagram summarizes the main metabolic outcomes promoted by in vivo administration of: (1) capsaicin, (2) resveratrol, (3) epigallocatechin-3-gallate, and (4) quercetin. Despite their limited absorption and low bioavailability, these compounds consistently improve diverse metabolic outcomes. On the other hand, they also modulate the colon microbiota, albeit the currently available data do not support yet whether such modulation is a feasible mechanism of action for their metabolic properties. HDL, high-density lipoprotein; LDL, low-density lipoprotein; VLDL, very-low-density lipoprotein; GLU, blood glucose; INS, insulin; DNL, de novo lipogenesis, TAG, triacylglycerols; SBP, systolic blood pressure; DBP, diastolic blood pressure; MBP, mean blood pressure; WAT, white adipose tissue; BW, body weight. The arrows imply the kinetics routes; question mark (?) implies lack of consistent data to implicate the respective pathway in the displayed metabolic outcomes. The diagram has been created with BioRender.com.

Plant-derived secondary metabolites have played a principal role in evidence-based ethnopharmacology and importantly driven drug design and development strategies. In particular, (poly)phenolic compounds, as demonstrated above, are undoubtedly able to promote endocrine homeostasis through the improvement of diverse metabolic outcomes (Figure 1). It is intriguing how (poly)phenols sustain such metabolic efficacy in spite of their very low and broadly variable gastrointestinal absorption [27]. The resolution of this puzzle widely depends on the availability of the pharmacokinetics data of (poly)phenols which are still scarce. For instance, only few studies amongst the 32 reports scrutinized here have assessed the blood levels of their respective phenolic compounds. Biotechnological approaches have been seen as important tools to improve (poly)phenols bioavailability. However, as recently reported, the impact of technological and biotechnological processes on the bioavailability of different families of phenolic compounds in humans has been minimally studied so far [103].

Last but not least, despite their widely propelled prebiotic properties, the available body of evidence still does not support the modulation of the gut microbiota growth and metabolism as a main mechanism of action (Figure 1). The data showed in Table 1 clearly demonstrate that the gut microbiota response to a given (poly)phenol lacks a characterizing pattern even at the phylum level. Regarding the triggering of gut microbiota-derived biochemical and molecular signaling pathways by (poly)phenols, available data are yet more scarce. For instance, SCFA have been ascribed as main mediators of (poly)phenols metabolic outcomes [21,22,23,104,105,106], although the literature fails to provide supportive data, as recently showed by our group [107]. On the other hand, it is worthy to mention that (poly)phenols modulatory role on the gut microbiota might be due the synergy between their prebiotic and antimicrobial properties, which was recently defined as “duplibiotic effect” [24]. Thus, it is difficult to translate basic science research into medical interventions mostly due to the large compositional complexity of the microbiome, which results in datasets that still need sophisticated statistical methods for their analysis.

## 4. Closing Remarks

Overall, the studies discussed in this review show that diverse factors, such as dietary pattern, gender, ethnicity, and genetic background among others, influence gut microbiota response in different ways, which causes the effectiveness of a particular (poly)phenol to vary according to the microbiome profile of each individual. This assumption is corroborated by the European MetaHIT data, which demonstrated that gut microbiota contains only around 160 out of 1000–1150 possible commensal species [36]. Thus, despite the consistent evidence supporting the metabolic properties of (poly)phenols, scarce data corroborate the modulation of the gut microbiota as a leading role. As recently highlighted by Brussow [41], microbiome research and its related interventional studies are mostly in a descriptive phase, which still wait for the harmonization of modulatory patterns in a way that adequately guides the assessment of experimental and clinical approaches. Nevertheless, the increasing interest in microbiota-targeted nutraceutical strategies, allied to emerging technologies, will open future lines of investigation to assess the individual microbiota profile. This will be of particular importance as a supportive tool for the prescription and follow-up of patients under prebiotic (poly)phenols regimens.

## Figures and Tables

**Table 1:** Modulatory effects of plant-derived (poly)phenols on the gut microbiota and their metabolic outcomes.

(Poly)phenol	Model	Regimen	Gut Microbiota Modulation	Metabolic Outcomes	Ref.
**Pre-clinical Studies**					
Resveratrol	DSS-induced colitic rats	1 mg/kg/day supplemented to the diet for 25 days.	↑ *Bifidobacterium* , ↓ *Enterococcus faecalis*, ↑ *Lactobacillus*.	↑ food intake, ↓ body weight loss associated to the animal model.	[64]
	HFD-fed mice	60 mg/kg/day supplemented to the diet for 5 weeks.	↓ *Bacteroides vulgatus*, ↓ *Alistipes putredinis*, ↓ *Parabacteroides johnsonii*.	↑ glucose tolerance, ↑ GLP-1 and insulin levels, ↑ GLP-1 intestinal content.	[65]
	HFD-fed mice	200 mg/kg/day supplemented to the diet for 12 weeks.	↑ Bacteroidetes, ↑ *Bifidobacterium*, ↑ *Lactobacillus*, ↓ *Enterococcus faecalis*.	↓ body weight, ↓ abdominal adipose tissue, ↓ liver weight, ↓ glycemia, ↓ dyslipidemia.	[66]
	HFD-fed mice	200 mg/kg/day by oral gavage for 8 weeks.	↓ *Lactococcus*, ↓ *Oscillibacter*, ↓ *Clostridium XI*, ↓ *Flavonifractor*, ↓ *Hydrogenoanaerobacterium*.	↓ body weight, ↓ epididymal adipose tissue, ↑ glucose tolerance, ↑ insulin sensitivity.	[67]
	HFHS-fed mice	400 mg/kg/day supplemented to the diet for 8 weeks.	↑ *Bacteroides,* ↓ *Turicibacteraceae*, ↓ *Lachnospiraceae*, ↑ *Parabacteroides,* ↓ *Akkermansia*.	↓ fat mass, ↑ glucose tolerance.	[68]
	HFD-fed mice	400 mg/kg/day supplemented to the diet for 4 weeks *.	↑ *Lactobacillus,* ↑ *Bifidobacterium*, ↓ Proteobacteria.	↓ body weight gain, ↑ glucose homeostasis, ↓ perigonadal and inguinal adipose tissue, ↑ white adipose tissue browning.	[69]
	HFD-fed mice	400 mg/kg/day supplemented to the diet for 16 weeks.	↑ *Erysipelotrichaceae* family, ↑ *Allobaculum spp.*	↓ body weight, ↓ subcutaneous and visceral adipose tissue, ↑ lean mass, ↓ food intake.	[70]
	Fecal transplantation from HFD-fed RSV-treated to HFD-fed untreated mice	300 mg/kg/day by oral gavage for 16 weeks.	↑ *Bacteroides*, ↑ *Lachnospiraceae*, ↑ *Lachnoclostridium*, ↑ *Parabacteroides*, ↑ *Ruminiclostridium,* ↑ *Blautia*.	↓ body weight, ↓ white adipose tissue, ↑ white adipose tissue browning, ↓ blood glucose, ↑ insulin sensitivity, ↓ hepatic steatosis, ↓ serum LPS levels.	[71]
	Perinatal and post-weaning HFr-fed rats	50 mg/L in drinking water to mothers and offspring up to 12 weeks old.	↓ Bacteroidetes, ↑ *Lactobacillus*, ↑ *Bifidobacterium*, ↑ *Akkermansia.*	↑ body weight, ↓ blood pressure, ↓ renal oxidative stress, ↑ nutrient-sensing signals.	[72].
	HFD-fed mice	60 mg/kg/day supplemented to the diet for 5 weeks.	↓ *Rikenellaceae,*↑ *Ruminococcaceae*, ↓ *Peptostreptococcaceae,* ↓ Proteobacteria.	↑ glucose tolerance.	[73]
	HFD-fed rats	10 mg/kg/day supplemented to the diet for 8 weeks.	↓ *Bacteroides,* ↑ *Lachnospiraceae*, ↓ *Desulfovibrionaceae.*	↓ blood glucose, ↑ insulin sensitivity.	[74].
	HFHFr-fed rats	30 mg/kg/day supplemented to the diet for 8 weeks.	↑ *Blautia*, ↑ *Moryella*, ↑ *Lactococcus*.	↓ liver weight, ↓ hepatic transaminases levels, ↓ steatohepatitis.	[75]
Pterostilbene	Obese Zucker (*fa/fa*) rats	15 mg/kg/day by oral gavage for 6 weeks.	↑ *Mollicutes*, ↓ *Negativicutes*, ↓ *Lachnospiraceae*, ↓ *Defluviitaleaceae*, ↑ Verrucomicrobia.	↓ body weight gain, ↓ white adipose tissue, ↓ insulin levels, ↑ insulin sensitivity.	[76]
	HFHF-fed rats	15 and 30 mg/kg/day supplemented to the diet for 8 weeks.	↓ *Clostridium* sensu stricto 1, ↑ *Erysipelatoclostridium*, ↑ *Fourrnierella*, ↑ *Akkermansia*.	↓ hepatic transaminases levels, ↓ steatohepatitis.	[75]
EGCG	Wistar rats	300 and 600 mg/kg/day supplemented to the diet for 4 weeks *.	↑ *Bacteroides,* ↓ *Prevotella*, ↓ *Clostridium*, ↓ *Bifidobacterium*.	↓ liver weight, ↓ abdominal adipose tissue (higher dose).	[77]
	ICR mice	50, 750, or 1500 mg/kg/day supplemented to the diet for 2–10 days *.	↓ *Clostridium* cluster IV, ↓ *Clostridium* cluster XIVa.	↓ CYP3A gene and protein expression in the liver, ↓ pregnane X receptor (PXR) protein expression in the liver (higher dose).	[78]
	HFD-fed mice	320 mg/kg/day (roughly) supplemented to the diet for 8 weeks *.	↑ *Allobaculum*, ↑ *Clostridium*, ↑ *Parabacteroides*, ↓ *Lachnospiraceae*, ↓ *Ruminococcous*, ↑ *Adlercreutzia*, ↓ *Desulfovibrionaceae*, ↑ *Akkermansia*.	↓ body weight, ↓ hepatic steatosis, ↓ hepatic TG, ↓ serum non-esterified fatty acids.	[79]
Quercetin	HFD-fed mice	50 mg/kg/day aglycone quercetin supplemented to the diet for 16 weeks *.	↑ *Bacteroidia**,*↑ *Erysipelotrichi*, ↓ *Bacilli*, ↓ *Clostridia*, ↓ *Helicobacter*, ↑ *Betaproteobacteria*, ↓ *Desulfovibrio*, ↓ *Deltaproteobacteria*, ↑ *Akkermansia*.	↓ body weight gain, ↓ epididymal fat pads, ↓ glycemia, ↓ insulinemia, ↑ insulin sensitivity, ↓ plasma TG, ↓ plasma alanine aminotransferase activity, ↓ hepatic steatohepatitis.	[80]
Hesperetin	Wistar rats	500 mg/kg/day supplemented to the diet for 3 weeks *.	↓ *Clostridium* subcluster XIVa, ↑ *Clostridium* clusters IV, XVIII.	↓ abdominal adipose tissue.	[81]
Theaflavins	*db/db* mice	100 mg/kg/day supplemented to the diet for 7 weeks *.	↓ *Barnesiella,* ↓ *Odoribacter*, ↓ *Lachnospiraceae*, ↓ *Desulfovibrio*.	↓ insulinemia.	[82]
Sinapine	HFD-fed mice	500 mg/kg/day supplemented to the diet for 12 weeks *.	↑ *Prevotellaceae,*↑ *Lactobacillaceae*, ↓ *Lachnospiraceae*, ↓ *Erysipelotrichaceae*, ↓ *Peptostreptococcaceae*, ↑ *Blautia*, ↑ *Bifidobacterium*, ↑ *Eggerthellaceae*, ↓ *Desulfovibrio*, ↑ *Akkermansiaceae*.	↓ body weight, ↓ food efficiency, ↓ white adipose tissue, ↓ blood glucose, ↓ plasma TG, ↓ plasma LDL-C, ↓ insulinemia, ↑ insulin sensitivity, ↓ hepatic steatosis.	[83]
Hydroxytyrosol	Fine particulate matter-exposed mice	50 mg/kg/day by oral gavage for 4 weeks.	↑ Bacteroidetes, ↑ *Akkermansia*.	↓ visceral adipose tissue, ↑ glucose tolerance, ↑ insulin sensitivity, ↓ hepatic oxidative stress, ↓ hepatic inflammation.	[84]
	HFD-fed mice	50 mg/kg/day by oral gavage for 8 weeks.	Unchanged Bacteroidetes/Firmicutes.	↓ white adipose tissue, ↓ liver weight, ↓ blood glucose, ↑ insulin sensitivity, ↓ hepatic steatosis, ↓ plasma LPS.	[85]
Capsaicin	HFD-fed mice	2 mg/kg/day supplemented to the diet for 12 weeks *.	↓ LPS-producing S24-7 family, ↑ *Ruminococcaceae*, ↑ *Lachnospiraceae*.	↓ body weight gain, ↓ white adipose tissue, ↑ glucose tolerance, ↓ serum LPS, ↓ serum proinflammatory cytokines.	[86]
	HFD-fed mice	10 mg/kg/day supplemented to the diet for 9 weeks *.	↑ *Bacteroides*, ↑ *Prevotella*, ↑ *Coprococcus*, ↑ *Akkermansia*, ↓ Proteobacteria, ↑ Acidobacteria.	↓ body weight gain, ↓ food intake, ↑ glucose tolerance.	[87]
	*ob/ob* mice	6 and 12 mg/kg/day supplemented to the diet for 6 weeks *.	↓ *Bacteroides*, ↑ *Roseburia*, ↑ *Parabacteroides*.	↑ glucose tolerance, ↑ insulin sensitivity.	[88]
	*db/db* mice	10 mg/kg/day added to the diet for 4 or 8 weeks *.	↓ *Lactobacillus*.	↓ blood glucose, ↓ insulinemia, ↑ glucose tolerance, ↑ insulin sensitivity.	[89]
	HFD-fed TRPV1^−/−^ mice	2 mg/kg/day by oral gavage for 12 weeks.	↑ *Bacteroides*, ↑ *Prevotella*, ↓ endotoxemic S24-7 family, ↑ *Coprococcus*, ↓ Actinobacteria, ↓ *Desulfovibrio*, ↓ *Escherichia*, ↓ *Helicobacter*, ↓ *Sutterella*, ↑ *Akkermansia*, ↓ Cyanobacteria, ↑ Tenericutes.	↓ body weight gain, ↓ food intake, ↓ blood glucose, ↓ plasma TG, TC, and LDL-C, ↓ insulinemia.	[90]
Dihydrocapsiate	HFD-fed mice	2 and 10 mg/kg/day by oral gavage for 12 weeks.	No change in *Lactobacillus*, *Bifidobacterium*, and *Akkermansia*.	↓ plasma TG, ↓ insulinemia, ↑ glucose tolerance, ↓ hepatic steatosis.	[91]
Honokiol	HFD-fed mice	200, 400 and 800 mg/kg/day supplemented to the diet for 8 weeks.	↑ *Bacteroides*, ↓ *Muribaculaceae*, ↓ *Oscillospira*, ↓ *Ruminococcus*, ↓ *Lactococcus*, ↓ *Dehalobacterium*, ↓ Unclassified_*Clostridiales*, ↓ Unclassified_*Ruminococcaceae*, ↑ Unclassified_*Enterobacteriaceae*, ↑ *Bilophila*, ↑ *Akkermansia*, ↑ *Fusobacterium.*	↓ body weight, ↓ white adipose tissue, ↓ serum TG, and TC, ↓ serum free fatty acids, ↓ blood glucose.	[92]
**Clinical Studies**					
Trans-resveratrol	MetS humans	2 g/day orally for 30 days.	↓ *Rikenellaceae*, ↓ *Butyricimonas,* ↑ *Gemellaceae*, ↑ *Turicibacter*, ↓ *Ruminococcus*, ↓ *Oscillospira*, ↓ *Clostridium*, ↓ *Odoribacter*, ↓ *Alistipes*, ↑ *Gammaproteobacteria*, ↑ *Akkermansia*, ↑ *Atopobium.*	↑ glucose tolerance in Caucasian subjects only.	[93]
Trans-resveratrol + EGCG	Overweight humans	80 mg/day RVS and 282 mg/day EGCG orally for 12 weeks.	↓ *Faecalibacteriuim prausnitzii*, ↓ Bacteroidetes (only in men).	↑ skeletal muscle mitochondrial oxidative capacity, ↑ increased fat oxidation.	[94]
Capsaicin	Humans	0.078 mg/kg/day for 2 weeks, 1 week washout and then 0.156 mg/kg/day for 2 weeks *.	↑ *Lachnospiraceae*, ↑ *Ruminococcaceae*, ↑ *Faecalibacterium*.	↑ plasma GLP-1, ↑ GIP and ghrelin.	[95]

Phyla to which mentioned bacteria families, genera or species pertain to are color-coded as: Bacteroidetes (blue), Firmicutes (red), Actinobacteria (purple), Proteobacteria (orange), Verrucomicrobia (green), and others (black). Abbreviations: DSS—dextran sulfate sodium; GLP-1—glucagon-like peptid-1; GIP—gastric inhibitory polypeptide; TG—triglyceride; TC—total cholesterol; HDL-C—high density lipoprotein cholesterol; LDL-C—low density lipoprotein cholesterol; HOMA—homeostatic model assessment; CYP3A—enzyme cytochrome P450 3A; LPS—lipopolysaccharide; MetS—metabolic syndrome; HFr—high fructose diet; HFD—high fat diet; HFHFr—high fat/high fructose; HFHS—high fat/high sugar; EGCG—epigallocatechin-3-gallate. ↑, implies increased levels or improved function; ↓, implies decreased levels or impaired function; *, implies that dose values were estimated from the consumption of the (poly)phenol-containing diet.
